# Money for value: the Kinzigtal-way to measure the produced value and health gain in a local area

**Published:** 2012-09-04

**Authors:** Helmut Hildebrandt

**Affiliations:** CEO OptiMedis AG and CEO Gesundes Kinzigtal GmbH, Germany

**Keywords:** preventive and health promotion programmes, trans-sector organisation, Gesundes Kinzigtal, population-based integrated care, integrated care pilot, Germany

## Abstract

‘Triple Aim’ is the buzzword for the initiatives of the Obama-administration in the US and is referring to a famous article of Don Berwick et al. in 2008 in health affairs asking for better health, better health care, and lower per capita costs. A similar venture started already in 2006 in Germany. One of the most challenging ventures towards reorienting health care in the direction of outcome-orientation is the measurement of the produced value and health gain in a local area. In this keynote the financial architecture and the specific way to measure the produced value and health gain in the integrated care pilot ‘Gesundes Kinzigtal’ will be described—as well some of the operating details and the results within.

Located in Southwest Germany, Gesundes Kinzigtal is the only population-based integrated care approaches in Germany, organising care across all health service sectors and indications, that is thoroughly scientifcally evaluated on its medical outcomes in regard to normal care. The system serving nearly half of the population of the region is run by a regional health management company in cooperation with the physicians’ network in the region, a German health care management company with a background in medical sociology and health economics and with two statutory health insurers.

Having started in 2006 the more effective trans-sector organisation of the local health care system and increased investments in well-designed preventive and health promotion programmes have led to a reduction in morbidity and mortality, and in particular to reduced overall costs for the insurees of these sickness funds. The results for one of the insurers show a substantial morbidity adjusted efficiency gain already for the years 2007–2010, rising to more than 16% of total costs (included are pharmaceutical, hospital, nursing, emergency as well as physiotherapist and sick leave costs).

More Information can be found on www.optimedis.de and www.gesundes-kinzigtal.de and on the evaluation (in German and English) www.ekiv.org.

